# Astragaloside IV Improves Bleomycin-Induced Pulmonary Fibrosis in Rats by Attenuating Extracellular Matrix Deposition

**DOI:** 10.3389/fphar.2017.00513

**Published:** 2017-08-08

**Authors:** Liu-Cheng Li, Liang Xu, Yan Hu, Wen-Jie Cui, Wen-Hui Cui, Wen-Cheng Zhou, Lian-Di Kan

**Affiliations:** ^1^Department of Pharmacy, Sir Run Run Shaw Hospital, School of Medicine, Zhejiang University Hangzhou, China; ^2^The First Affiliated Hospital of Anhui Medical University Hefei, China; ^3^Department of Emergency Medicine, Second Affiliated Hospital, School of Medicine, Zhejiang University Hangzhou, China; ^4^School of Pharmacy, Anhui Medical University Hefei, China

**Keywords:** pulmonary fibrosis, bleomycin, astragaloside IV, extracellular matrix, high-mobility group box1

## Abstract

Pulmonary fibrosis is a devastating lung disorder with mysterious pathogenesis and limited treatment options. It is well-recognized that the uncontrolled proliferation of lung fibroblasts and differentiation of fibroblasts into myofibroblasts excessively produce extracellular matrix (ECM) proteins which contribute to the fibrosis change of the lungs. Thus, blocking ECM accumulation would delay fibrosis progression. In this study, we observed the effects of astragaloside IV (ASV) (10 mg/kg/d) on ECM proteins in bleomycin (BLM, 5 mg/kg)-treated rats. Our results showed that ASV not only ameliorated BLM-induced body weight loss, lung coefficient increase, histological changes and collagen secretion, but also reduced the levels of type III collagen (Col-III) in lung homogenate, laminin (LN) and hyaluronic acid (HA) in serum, as well as hydroxyproline (HYP) in lung tissue. Besides, ASV significantly down-regulated the levels of high-mobility group box1 (HMGB1) in serum and lung tissue, and inhibited the up-regulated expression of α-SMA (marker of myofibroblasts) in the lungs. Taken together, these findings indicate that ASV attenuates BLM-induced ECM deposition, supporting its use as a promising candidate to treat lung fibrosis.

## Introduction

Pulmonary fibrosis (PF), characterized by exaggerated accumulation of extracellular matrix (ECM) proteins, is a progressive and irreversible fatal lung disease with a median survival time less than 3 years (King et al., 2011; Warsinske et al., 2016; Li and Kan, 2017). Although the etiology of PF is still unclear, emerging studies have shown that some special type of cells and cytokines play vital roles in the process of PF (Li et al., 2014b; Zhou et al., 2016). Several treatment strategies including the anti-fibrinolytic composition, glucocorticoids, and anti-oxidants have been proved to be effective in the laboratory studies or human PF. However, the medications such as glucocorticoids and immunosuppressive drugs have inevitably severe side effects and are not enough to improve patients’ prognosis (Behr, 2013). The hot new drugs pirfenidone is proved to have benefits for PF suffers, however, the effectiveness and safety for long-time use is an open question (Rogliani et al., 2016). Thus, exploring and developing new potential targets and agents to restrict PF are still urgently needed.

Considerable factors such as bleomycin (BLM), irradiation and paraquat have been demonstrated to trigger the persistent progress of PF through inducing fibroblasts proliferation and fibroblasts to myofibroblasts transformation, and result in excessive secretion of ECM proteins, and then lung consolidation and persistent fibrosis (Kendall and Feghali-Bostwick, 2014; Kirschner, 2016; Li and Kan, 2017). Thus, reversing or suppressing ECM deposition would be a pivotal strategy to restrict the progress of PF and the potential agents targeting ECM may provide novel treatment strategies (Rajasekaran et al., 2015). A lot of cytokines have been reported to induce the secretion of ECM proteins. High-mobility group box1 (HMGB1), a danger signal and inflammatory inducer, has been shown to directly stimulate fibroblast proliferation, and induce epithelial-mesenchymal transition (EMT) which is a critical mechanism in the acquisition of the power of mesenchymal cells (such as ECM-producing cells myofibroblasts) for alveolar epithelial cells, and promote the production of ECM proteins and contribute to PF (Hamada et al., 2008; Cai et al., 2015; Li et al., 2015a). Therefore, anti-HMGB1 agents may be an important option to block PF.

Herbal medicines as well as the extracts have been widely used to prevent or treat human diseases (Apaya et al., 2016; Li and Kan, 2017). Astragaloside IV (ASV), a new glycoside of cycloartane-type triterpene isolated from the medical herb *Astragalus membranaceus*, has multiple pharmacologic effects for its potent immunoregulatory, anti-inflammatory, anti-asthma, and anti-fibrotic actions (Meng et al., 2011; Li et al., 2017). It has shown that ASV protects against the progression of renal fibrosis, systemic sclerosis, liver fibrosis as well as myocardial fibrosis, all without evident toxicity or side effects (Chen et al., 2011; Li et al., 2013; Qi et al., 2014; Zhou et al., 2017). It reveals that ASV has special benefits on systemic sclerosis by decreasing collagen formation and suppresses collagen production of activated hepatic stellate cells (Li et al., 2013; Qi et al., 2014). Moreover, recent work has shown that ASV protects against BLM-induced PF by inhibiting the levels of oxidative stress and inflammatory response (Yu et al., 2016). However, the effects of ASV on the regulation of ECM proteins and lung myofibroblasts activation are still unclear. To bring new insights to this key question, we observed the effects of ASV on BLM-treated rats focused on evaluating its effects on ECM proteins and HMGB1 activity in this study. The data supported that ASV not only inhibited BLM-induced histological abnormalities but also down-regulated the levels of ECM proteins and HMGB1. Besides, it inhibited the protein expression of myofibroblasts marker α-smooth muscle actin (α-SMA). These *in vivo* observations suggest that ASV attenuates PF through inhibiting myofibroblasts proliferation and secretion of ECM proteins.

## Materials and Methods

### Antibodies and Reagents

The primary antibodies described in this paper including β-actin (ab52614, 1:5000; Abcam, Cambridge, MA, United States), HMGB1 (ab79823, 1:5000; Abcam, Cambridge, MA, United States), α-SMA (ab5694, 1:300; Abcam, Cambridge, MA, United States), goat anti-mouse lgG (ZB-2305, 1:10000; ZSGB-BIO, Beijing, China) and goat anti-rabbit lgG (ZB-2301, 1:10000; ZSGB-BIO, Beijing, China). In addition, the masson trichrome kit (Masson, MST-8003/8004, Maixin-Bio, China), hydroxyproline (HYP) assay kit (A030-2, Nanjing Jiancheng Bioengineering Institute, China), as well as other ELISA kits including HMGB1 (YY42027), type III collagen (Col-III, YY41621), hyaluronic acid (HA, YY42052) and laminin (LN, YY41730) were from Shanghai yuanye Bio-Technology Co., Ltd. (Shanghai, China) in this study.

### Animals

Female adult Sprague–Dawley rats were from the Laboratory Animal Center of Anhui Medical University, Hefei, China. All of the animals received standard food and water *ad libitum* during the research. The rats were acclimatized in the standard animal room for 3 days prior to starting the experiment. The experimental procedures were approved by the Institutional Animal Care and Use Committee at Anhui Medical University in accordance with the National Institutes of Health Guidelines for the Care and Use of Laboratory Animals.

### Drugs

Astragaloside IV was manufactured by Nanjing Spring & Autumn Biological Engineering, Co., Ltd., China (≥98%, 84687-43-4). BLM A5 hydrochloride (8 mg/vial, Laiboten Pharmaceutical, Co., Ltd., Harbin, China) was dissolved in 0.9% sodium chloride injection with a volume of 1.6 ml just before using. Sufficient ASV and prednisone (Pred) acetate (5 mg, Xinhua Pharmaceutical, Co., Ltd., Shandong, China) were respectively diluted with 0.5% carboxymethyl cellulose sodium (CMC-Na) solution just before gavage once a day.

### Establishment of Pulmonary Fibrosis Model

Eighty experimental rats were randomly assigned to the following groups including: (1) control group (Control), only instilled with saline; (2) BLM group (BLM), only instilled with BLM; (3) Pred group (BLM + Pred), instilled with BLM and treated with Pred (5 mg/kg); (4) ASV group (BLM + ASV), instilled with BLM and treated with ASV (10 mg/kg). The PF model was replicated by intratracheal instillation of BLM (5 mg/kg). Then Pred (5 mg/kg/d, dissolved in 0.5% CMC-Na) and ASV (10 mg/kg/d, dissolved in 0.5% CMC-Na) as well as equal volume of 0.5% CMC-Na (12 ml/kg) were respectively given to the rats in Pred group, ASV group, Control group and BLM group by gavage administration 1 day after BLM induction for 28 days. On days 14 and 28, the rats were weighed and intraperitoneally anesthetized with 10% chloral hydrate (2.5 ml/kg), subsequently, the lungs and blood were weighed or/and collected, stained with hematoxylin-eosin (HE) and Masson. Meanwhile, the levels of Col-III in lung homogenate, HMGB1, LN and HA in serum, as well as HYP in lung tissue were measured. Moreover, the protein expression of HMGB1 and α-SMA in the lungs was observed by western blot analysis.

### Histological Analysis

The left lung tissues were fixed in 10% formaldehyde for 48 h, dehydrated in graded ethanol and then embedded in paraffin. Sequential lung sections (5 μm) were for routine HE staining and Masson staining, respectively for pathologic analysis and locating collagen expression by using the standard protocols. The slides were obtained from a light microscope (Olympus Opticals, Tokyo, Japan) with the same magnification times (×200). The method details for the pathologic grades of alveolitis and fibrosis can be found in our previous work (Li et al., 2015b).

### Body Weight and Lung Coefficient Changes of Rats

On days 0, 7, 14 and 28, the body weight of experiment rats were weighed in each group. On the 14th and 28th days, the lungs of the sacrificed rats were collected and weighed. Then the lung coefficient was calculated as follows: lung coefficient = lung wet weight (g)/body weight (kg) × 100%.

### HYP, Col-III, LN, HA, and HMGB1 Levels Determination

As an indirect measure of the collagen level, HYP level in lung tissue was measured in concordance with the instruction manual of the HYP kit. The deputy lobes (80–100 mg) were used to detect the content of HYP which was expressed in microgram of HYP per milligram of wet weight (μg/mg). The lung homogenates were to measure Col-III content (ng/mL) by enzyme-linked immunosorbent assay (ELISA). The serum was to measure the levels of LN (ng/mL), HA (ng/mL), and HMGB1 (ng/mL) by ELISA. The absorbance in each samples were measured using an automated microplate reader at a wavelength of 550 nm (HYP) and 450 nm (Col-III, LN, HA, and HMGB1).

### Western Blot Analysis

Protein concentrations of the right lung lobes were determined in the supernatant of colonic tissues by classic BCA protein assay (Beyotime). Before the experiment, the loading buffer was mixed into the supernatant in a ratio of 1:4 and heated at 100°C for 10 min and equal protein amounts were separated by SDS-PAGE on a 12% gel and then transferred to polyvinylidene fluoride (PVDF) membranes (IPVH00010; Millipore, United States). Non-specific binding to the membrane was blocked for 2 h at room temperature with 5% non-fat dry milk (w/v) (Guangming, China) in TBST (AR0031, BOSTER, China) and incubated at 4°C overnight with the primary anti-HMGB1 (1:5000 diluted, 25 kDa), anti-α-SMA (1:300 diluted, 42 kDa), and anti-β-actin (1:5000 diluted, 43 kDa) antibodies. After washed with TBST, the blots were probed with horseradish peroxidase (HRP)-conjugated secondary antibodies (anti-rabbit immunoglobulin G (IgG) (ZSGB-BIO, Beijing, China) for 1 h at room temperature. Immunodetection was developed with enhanced chemiluminescence reagent (ECL, Beyotime, China). The experiment was performed independently at least three times. The densitometry was performed on protein bands using Image J analysis software (ChemiQ 4600, Bioshine, China). The integrated optical density (IOD) value was performed by Image-Pro Plus 6.0 and β-actin was used as an internal reference for relative quantification.

### Statistical Analysis

All data were presented as mean ± SD for each group. Difference among groups was performed by one-way ANOVA, followed by multiple comparisons using a *post hoc* LSD test or Dunnett’s T3 test. The scores of alveolitis and fibrosis were evaluated using the Mann–Whitney test. Statistical analysis was performed by SPSS 13.0 software, and *p*-values < 0.05 were considered to be statistically significant.

## Results

### ASV Improves BLM-Induced Histopathology Abnormalities

**FIGURE 1:**
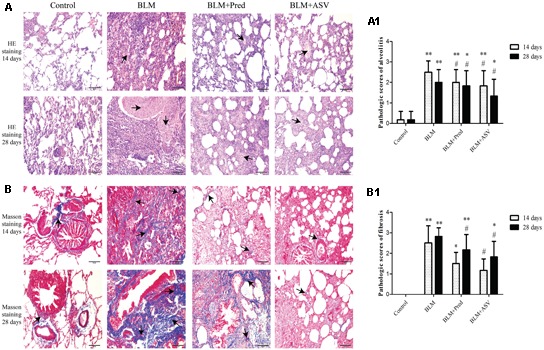
The pathologic changes in each group. **(A)** Typical images of HE staining, the arrows indicated the ECM proteins, ×200. **(A1)** The pathologic scores of alveolitis. **(B)** Masson staining showed the collagen expression in blue color (the arrows), ×200. **(B1)** The pathologic scores of fibrosis. The representative images were from the lungs in Control, BLM, BLM + Pred, and BLM + ASV animal groups on days 14 and 28. Six random visual fields of the sections from each group were evaluated in a blinded fashion for the pathologic grades of alveolitis and fibrosis. ^∗^*p* < 0.05 and ^∗∗^*p* < 0.01 vs. Control group; ^#^*p* < 0.05 vs. BLM group.

To evaluate BLM-treated pathologic changes and the effects of ASV, the histopathology abnormalities of the lungs in each group were first observed with HE staining. As shown in **Figure 1A**, BLM significantly induced the destruction of alveolar space, thickened the alveolar septa, and accelerated the production of superabundant ECM (especially on day 28) when compared with the Control group. Impressively, compared with BLM group, administration of Pred or ASV could attenuate the degree of PF to a certain degree (**Figure 1A1**), while the whole alveolar number in ASV group was more than that in Pred group.

### ASV Inhibited BLM-Induced Production of Collagen

Collagen is one of the crucial part of ECM which was mainly produced by myofibroblasts and directly contribution to PF (Knudsen et al., 2017). Here, Masson staining was performed to observe the expression of collagen in the lung tissue. After BLM administration, a large amount of collagen was accumulated in the lung tissue (the blue area as indicated in **Figure 1B**), while it was precious few in the Control group. In ASV group, ASV could markedly lower BLM-induced collagen expression compared to Pred group and BLM group. The scores also showed the protective role of ASV on the fibrosis changes (**Figure 1B1**).

### ASV Reduced Rats’ Lung Coefficient and Increased Body Weight

**FIGURE 2:**
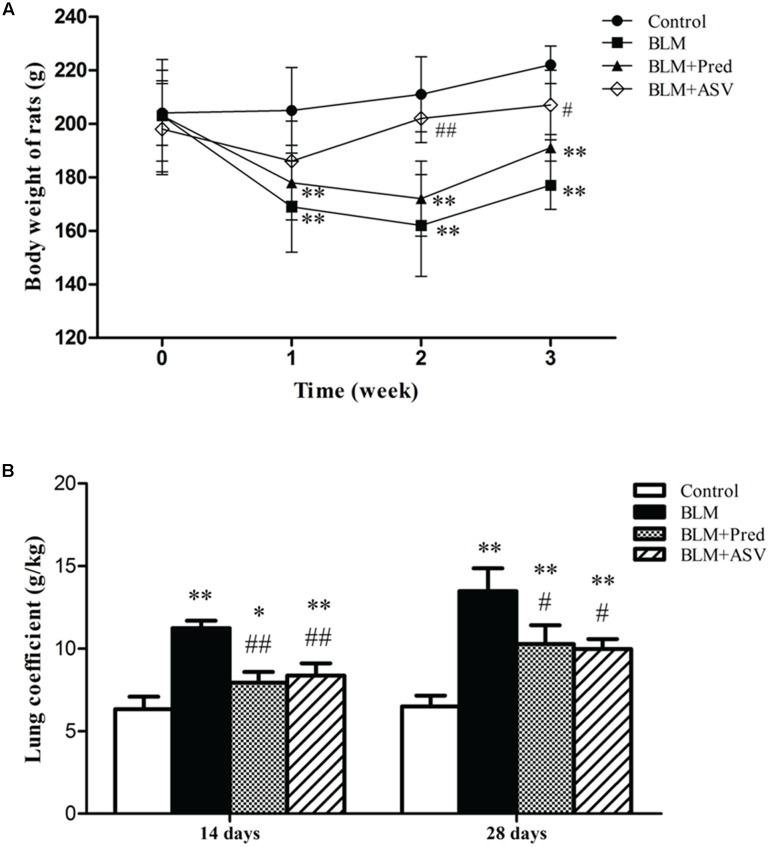
Effects of ASV on the body weight and lung coefficient. **(A)** The changed body weight of the rats on days 0, 7, 14, and 28. **(B)** The changes of lung coefficient in each group. Data were presented as means ± SD (*n* = 10). ^∗^*p* < 0.05 and ^∗∗^*p* < 0.01 vs. Control group; ^#^*p* < 0.05 and ^##^*p* < 0.01 vs. BLM group.

Then we analyzed the effects of ASV on rats’ lung coefficient and body weight. The data showed that BLM administration evidently decreased the rats’ body weight on days 7, 14, and 28 (**Figure 2A**) and increased the rats’ lung coefficient on days 14 and 28 (**Figure 2B**) compared with the Control group. However, ASV treatment increased the rats’ body weight especially on days 14 (*p* < 0.01) and 28 (*p* < 0.05) compared with BLM group, whereas Pred had no benefit on the body weight loss induced by BLM (**Figure 2A**). As shown in **Figure 2B**, ASV and Pred significantly decreased the increase of lung coefficient induced by BLM.

### ASV Decreased the Levels of HYP and Col-III in the Lungs

**FIGURE 3:**
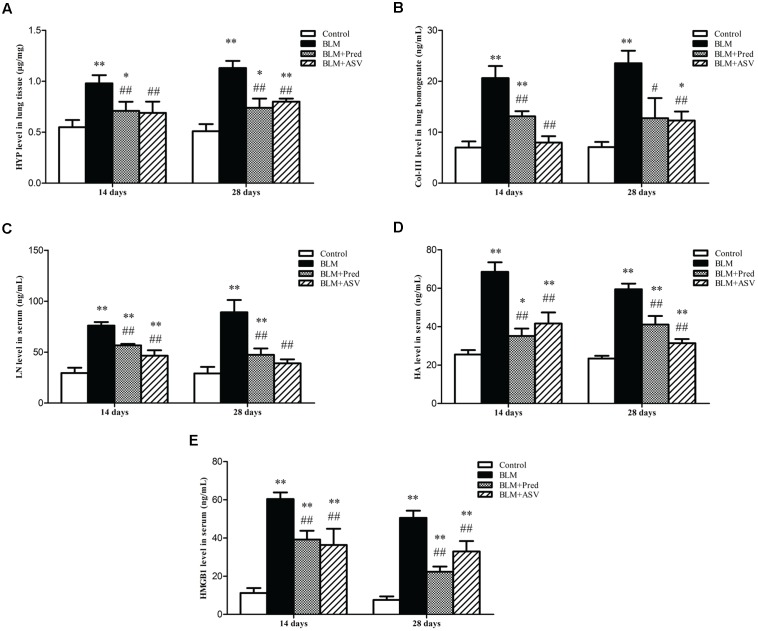
Effects of ASV on the levels of HYP, Col-III, LN, HA, and HMGB1. **(A)** The content of HYP in lung tissue. **(B)** The level of Col-III in lung homogenate. **(C)** The content of LN in serum. **(D)** The content of HA in serum. **(E)** The level of HMGB1 in serum. Data were presented as means ± SD (*n* = 10). ^∗^*p* < 0.05 and ^∗∗^*p* < 0.01 vs. Control group; ^#^*p* < 0.05 and ^##^*p* < 0.01 vs. BLM group.

Hydroxyproline is the main constituent of collagen, one kind of ECM proteins. The level of HYP could partly quantify the degree of PF. In this study, the HYP level in lung tissue was obviously reduced after treatment with ASV when compared with the BLM group (**Figure 3A**). Besides, BLM treatment significantly up-regulated the Col-III level in lung homogenate especially on day 28 (**Figure 3B**). Whereas ASV and Pred reduced Col-III level, especially that treated with ASV on both days compared with BLM group (*p* < 0.01). Combined with the Masson staining, these data further show the restricted effect of ASV on BLM-induced collagen production.

### ASV Reduced the Serum Levels of LN, HA, and HMGB1

HMGB1 plays a crucial role in EMT, activating fibroblasts and myofibroblasts which can directly produce ECM, and then promote lung consolidation and PF (Hamada et al., 2008; Li et al., 2015a). Other than collagen which had been previously investigated, LN and HA are also important parts of ECM which contribute to PF (Cui et al., 2015; Knudsen et al., 2017). In present work, we found that both ASV and Pred could significantly reduced BLM-induced increase of LN (**Figure 3C**), HA (**Figure 3D**), and HMGB1 (**Figure 3E**). It demonstrated that ASV restricted ECM production such as LN and HA, which may be partly due to the inhibited HMGB1 activity.

### ASV Down-regulated the Protein Expression of α-SMA and HMGB1 by Western Blot Analysis

**FIGURE 4:**
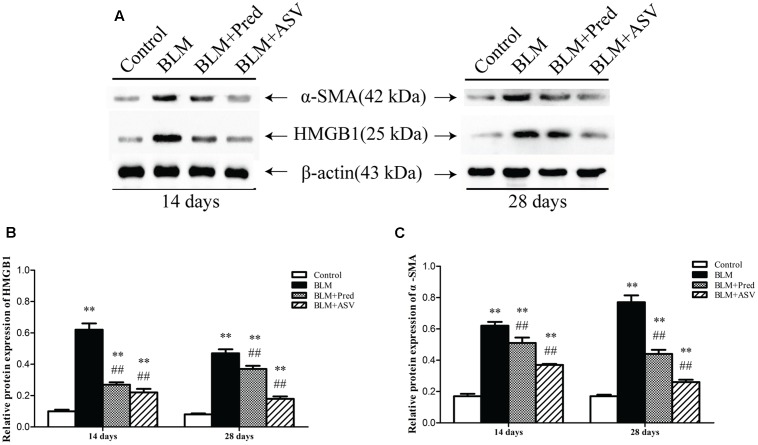
Effects of ASV on the expression of HMGB1 and α-SMA in the lungs. **(A)** Western blot of HMGB1 and α-SMA. **(B)** The relative protein expression of HMGB1. **(C)** The relative protein expression of α-SMA. The densitometry values were normalized to β-actin. Histogram bars represent mean ± SD of at least three independent experiments. ^∗∗^*p* < 0.01 vs. Control group; ^##^*p* < 0.01 vs. BLM group.

α-SMA is a key marker of myofibroblasts and thus its expression can reflect the activity of these ECM-producing cells. As shown in **Figure 4**, it showed little expression of α-SMA and HMGB1 in the Control group, while BLM treatment led to markedly up-regulated expression of them by western blot analysis. However, in ASV-treated group, the increased expression of HMGB1 (**Figure 4B**) and α-SMA (**Figure 4C**) was dramatically blocked compared to BLM group (*p* < 0.01). These results revealed the inhibitory role of ASV on myofibroblasts (α-SMA positive) proliferation and HMGB1 activation.

## Discussion

Pulmonary fibrosis is a chronic progressive and eventually fatal interstitial lung diseases with an unclear etiology, a poor prognosis and limited therapies. One of the pivotal hallmarks of PF are aberrant proliferation and activation of fibroblasts and myofibroblasts accompanied by excessive production of ECM (collagen, LN, HA, etc.) (Kendall and Feghali-Bostwick, 2014; Pardo and Selman, 2016). Up to now, a lot of work has been conducted to illustrate the potential drugs for PF treatment. However, limited effective candidates are discovered. Therefore, researches identifying innocuous anti-fibrotic agents are of high priority and urgently needed. ASV is one of the most active compounds isolated from *Radix Astragali*, an edible herb used widely in traditional Chinese medicine for several centuries (Sun et al., 2010). ASV has been used to treat several fibrotic disease *in vivo* including systemic sclerosis, liver fibrosis as well as lung fibrosis (Li et al., 2013; Qi et al., 2014; Yu et al., 2016). However, its role on several ECM proteins and myofibroblasts proliferation in lung fibrosis is unknown yet.

In this study, we first verified the success of PF model in rats from the distinctive pathology pictures. Our results demonstrated that ASV suppressed BLM-mediated destruction of alveolar structure, and reversed ECM accumulation as well as collagen expression in the lung. It may due to the benefits of ASV on BLM-induced abnormal alveolar structure by inhibiting ECM deposition. Furthermore, we found BLM treatment obviously decreased rats’ body weight and increased lung coefficient, which were reversed in ASV group, further indicating that ASV improved the general condition of the BLM-treated rats and reduced the lung consolidation. However, Pred, as the positive control drug, was showed to protect lung structure and reduce BLM-induced collagen expression to some degree when compared with BLM group, but it has no evident effect on the change of the rats’ body weight, which may be related to the side effects of Pred with restricted application in clinic (Behr, 2013). Then, we found that ASV evidently reduced the increase of HYP, Col-III, LN, and HA which demonstrated that ASV reversed ECM proteins secretion in BLM treated rats. Besides, we found that ASV could inhibit the expression of α-SMA, a key marker of lung myofibroblasts, supporting that ASV might reduce myofibroblasts activation after BLM administration.

**FIGURE 5:**
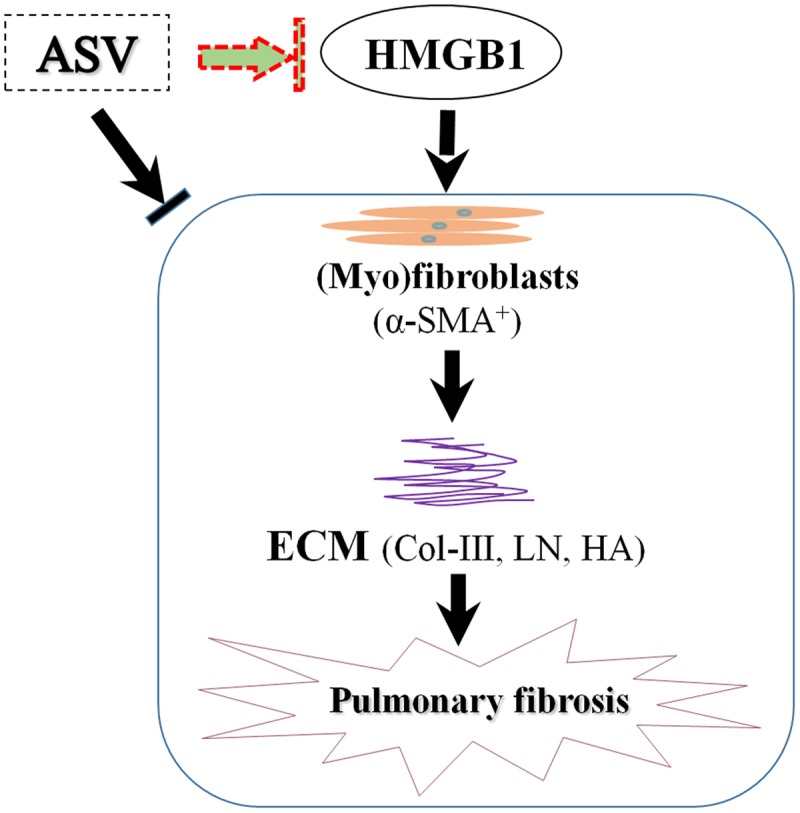
The potential mechanism of ASV on pulmonary fibrosis in rats. The protective role of ASV may partly due to the reduced release of HMGB1 with the inhibition of myofibroblasts (α-SMA^+^) proliferation and ECM deposition.

From recent studies, HMGB1 activity is essential in fibroblasts proliferation, EMT, eventual ECM deposition (Hamada et al., 2008; Li et al., 2014a, 2015a). We here found that the serum content and lung level of HMGB1 were significantly up-regulated after BLM administration. In contrast, we observed that ASV treatment counteracted the expression of HMGB1, suggesting that the anti-fibrotic role of ASV is at least partly due to the inhibition of HMGB1 release. Our results reveal that the protective role of ASV may partly due to the reduced release of HMGB1 with the inhibition of myofibroblasts proliferation and ECM deposition (**Figure 5**). Previous work has also shown that ASV ameliorates oxidative stress and inflammation in the lung (Yu et al., 2016). Take together, the therapeutic effects of ASV are based upon a combination of anti-oxidation, anti-inflammation in the early stage of lung injury, as well as the decrease of myofibroblasts proliferation and ECM deposition in the late stage during fibrosis. However, more detailed investigations are urgent needed to prove the role of ASV on fibroblasts and alveolar epithelial cells *in vitro*, and its regulation on HMGB1 related signaling pathway and EMT.

## Conclusion

This study demonstrated the anti-fibrotic effects of ASV in lung fibrosis, which may be due to its regulation on HMGB1 release and ECM production, suggesting an attractive pharmacological tool for the treatment of PF.

## Author Contributions

Conceived and designed the experiments: L-CL, LX. Performed the experiments: L-CL, LX, W-HC, and W-CZ. Contributed reagents, materials and analysis tools: L-CL, YH, W-JC, and L-DK. Analyzed the data: L-CL, YH, and W-JC. Wrote or modified the paper: L-CL, W-JC, and L-DK. All authors contributed to and approved the final draft of the manuscript.

## Conflict of Interest Statement

The authors declare that the research was conducted in the absence of any commercial or financial relationships that could be construed as a potential conflict of interest.
